# Differential genome organization revealed by comparative topological analysis of *Mycobacterium tuberculosis* strains H37Rv and H37Ra

**DOI:** 10.1128/msystems.00562-24

**Published:** 2025-04-07

**Authors:** Mohit Mishra, Ajay Arya, Md. Zubbair Malik, Akanksha Mishra, Seyed E. Hasnain, Rakesh Bhatnagar, Shandar Ahmad, Rupesh Chaturvedi

**Affiliations:** 1 School of Biotechnology, Jawaharlal Nehru University, New Delhi, India; 2 School of Computational and Integrative Sciences, Jawaharlal Nehru University, New Delhi, India; 3 Department of Genetics and Bioinformatics, Dasman Diabetes Institute, Dasman, Kuwait City, Kuwait; 4 Department of Biochemical Engineering and Biotechnology, Indian Institute of Technology Delhi, New Delhi, India; 5 Department of Life Science, School of Basic Sciences and Research, Sharda University, Greater Noida, India; 6 Amity University, Jaipur, Rajasthan, India; 7 Special Center for System Medicine, Jawaharlal Nehru University, New Delhi, India; 8 Nanofluidiks Pvt. Ltd, Jawaharlal Nehru University-Foundation for Innovation, New Delhi, India; Vanderbilt University Medical Center, Nashville, Tennessee, USA

**Keywords:** *M. tuberculosis*, Hi-C, CID, CID boundaries, gene expression

## Abstract

Recent studies have shown that three-dimensional architecture of bacterial chromatin plays an important role in gene expression regulation. However, genome topological organization in *Mycobacterium tuberculosis*, the etiologic agent of tuberculosis, remains unknown. On the other hand, the exact mechanism of differential pathogenesis in the canonical strains of *M. tuberculosis* H37Rv and H37Ra remains poorly understood in terms of their raw sequences. In this context, a detailed contact map from a Hi-C experiment is a candidate for what bridges the gap. Here, we present the first comprehensive report on genome-wide contact maps between regions of H37Rv and H37Ra genomes. We tracked differences between the genome architectures of H37Rv and H37Ra, which could possibly explain the virulence attenuation in H37Ra. We confirm the existence of a differential organization between the two strains most significantly a higher chromosome interaction domain (CID) size in the attenuated H37Ra strain. CID boundaries are also found enriched with highly expressed genes and with higher operon density in H37Rv. Furthermore, most of the differentially expressed PE/PPE genes were present near the CID boundaries in H37Rv and not in H37Ra. We also found a systemic reorganization of CIDs in both virulent H37Rv and avirulent H37Ra strains after hypoxia induction. Collectively, our study proposes a differential genomic topological pattern between H37Rv and H37Ra, which could explain the virulence attenuation in H37Ra.

**IMPORTANCE:**

Genome organization studies using chromosome conformation capture techniques have proved to be useful in establishing a three-dimensional (3D) landscape of bacterial chromatin. The sequence-based studies failed to unveil the exact mechanism for virulence attenuation in one of the *Mycobacterium tuberculosis* strains H37Ra. Moreover, as of today, no study investigated the 3D structure of the *M. tuberculosis* genome and how 3D genome organization affects transcription in *M. tuberculosis*. We investigated the genome topology in virulent and attenuated strains of *M. tuberculosis* using Hi-C. Our study demonstrated that virulent and attenuated *M. tuberculosis* strains exhibit distinct topological features that correlate with higher gene expression of virulence genes in the virulent H37Rv strain.

## INTRODUCTION

Recent studies have unveiled that prokaryotic organisms feature very organized chromosome structures, which were hitherto discussed mainly for eukaryotic genomes (1–5). Among them, nucleoid-associated proteins (NAPs) help in maintaining the dynamic organization of the chromosome in the absence of histones in bacteria (6). Invention of chromosome conformation capture (3C) and its derivative techniques has opened up opportunities for investigating three-dimensional (3D) structures of bacterial chromosomes and its long-range impact on gene expression regulation (7). Recent studies aimed at elucidating bacterial chromosome organization have provided enough evidence suggesting the presence of chromosome interaction domains (CIDs) in bacteria, very similar to topologically associated domains (TADs) found in eukaryotic organisms (1, 3, 5). Combination of 3C and Hi-C technology has made it possible to investigate comprehensive genome-wide interactions in some bacterial species such as *Escherichia coli* (1), *Caulobacter crescentus* (3), and *Bacillus subtilis* (4).

*Mycobacterium tuberculosis* is an extraordinary pathogen that latently infects almost one-third of the human population and also becoming refractile to treatment with rapidly developing multi-drug resistance (8, 9). H37Rv and its attenuated counterpart H37Ra both are derived from the same parental strain H37 and have been widely used as laboratory strains for research aiming to understand *M. tuberculosis* pathogenesis (10). H37Ra resembles the H37Rv genome in terms of gene order and gene content; however, it is 8,445 bp longer because H37Ra has 21 deletions and 53 insertions compared with the H37Rv strain (11). Major genetic changes are also caused by the differences in the repetitive sequences like IS6110 and the genes belonging to the PE/PPE/PE-PGRS family (11). However, a study by Elghraoui et al*.* using SMRT genome assembly demonstrated that H37Ra is significantly more similar to H37Rv than indicated previously by the Sanger-based reference sequence H37RaJH with contradicted variants overrepresented in the PE_PPE genes (12). Although the specific variants have been shown to significantly account for virulence attenuation and much has been studied on their virulence, an exact mechanism of virulence attenuation in H37Ra is still not completely understood (13–15).

The attenuated H37Ra strain is obviously expected to exhibit some alterations to either the genome or differential gene expression of virulence genes as compared with the virulent H37Rv strain. The *M. tuberculosis* genome is estimated to be more than 4 Mb long with over 4,000 protein-encoding genes, including 170 transcription factors (TFs), numerous DNA binding proteins, and many sigma factors, each of which performing critical functions under various stress responses (16). How such a large number of functional entities are stuffed into this small genome remains an unresolved enigma.

By mapping a detailed contact organization in *M. tuberculosis*, using Hi-C, we aim to explore the genome organization in *M. tuberculosis*. Hi-C provides a two-dimensional map of complete 3D organization of chromatin in the form of pairwise genomic fragments data. In order to understand the 3D genome organization in *M. tuberculosis*, in this study, we have used exponentially growing mycobacterial culture. We performed Hi-C on both virulent and attenuated strains of *M. tuberculosis* and analyzed data by using HiCExplorer tools (17). We constructed genome-wide contact map at 10 kb resolution and observed that the Ori and the midpoint are located at the two opposite poles of the chromosome structure. A differential CID organization was observed between virulent and attenuated strains with larger CIDs in attenuated strain H37Ra as compared with virulent counterpart H37Rv. We proposed that it could be a factor for virulence attenuation in H37Ra. We also observed that most of the CID boundaries were enriched with known highly expressed genes. Interestingly, most of the genes belonging to PE-PPE family of genes with increased expression in H37Rv as compared with H37Ra are present near the CID boundaries in H37Rv. To find out whether a physiological perturbation such as hypoxia has an effect on chromosome organization in *M. tuberculosis*, we performed Hi-C on hypoxia-induced cultures of both H37Rv and H37Ra. We found a systemic reorganization of CIDs in both virulent H37Rv and avirulent H37Ra strains. Most of the CIDs in hypoxia-induced H37Rv were merged to form larger CIDs as compared with aerobic H37Rv. So collectively, this differential CID organization in virulent and attenuated strains could indeed provide a novel way of transcriptional gene regulation under aerobic and hypoxic conditions and could be one of the mechanisms for attenuation.

## RESULTS

### Comparative genome organization in virulent and attenuated *Mycobacterium tuberculosis* strains

To study chromosome organization in *M. tuberculosis*, we applied the Hi-C method to exponentially growing wild-type H37Rv and H37Ra cultures. A total of 75 million pairs of sequencing reads were generated for H37Rv, and 110 million pairs of reads were generated for H37Ra (Table S1). To analyze the contact information contained in them, we first mapped the resulting sequencing reads to the reference genome of H37Rv (NC 000962.3 NCBI) and H37Ra (NC_009525.1 NCBI) encompassing 4,411,532 bp and 4,419,977 bp, respectively. We obtained around 70 million high-quality mapped reads in H37Rv and around 100 million in H37Ra. Further comprehensive sequence analysis identified around 19 and 40 million valid read pairs in H37Rv (26%) and H37Ra (41%), respectively, for 3D genome construction.

Intra long-range interactions (≥20 kb) revealed by Hi-C were much more frequent than intra short-range interactions (<20 kb) in both H37Rv and H37Ra (Table S1). The genome-wide matrices were then constructed with valid Hi-C reads at 10 kb resolution representing the final interaction frequencies (*x*, *y*), which reflects the relative contact frequency between bins *x* and *y*. We confirmed that biological replicates were highly correlated (Pearson correlation coefficient > 0.92) in both H37Rv and H37Ra (Fig. S1).

**Fig 1:**
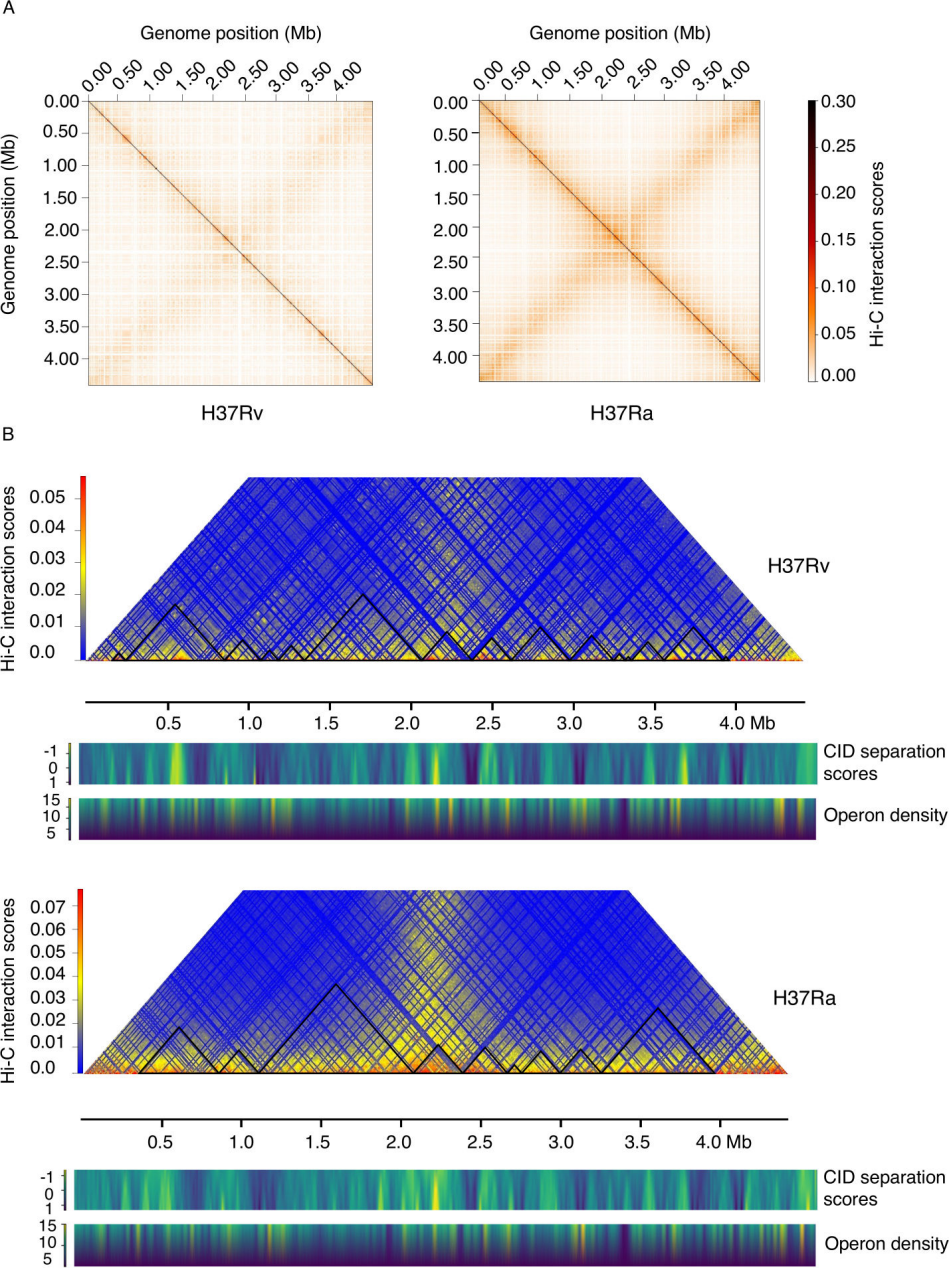
Comparative analysis of structural organization of circular chromosome in *Mycobacterium tuberculosis* strains H37Rv and H37Ra. (**A**) Normalized BglII Hi-C contact map of *M. tuberculosis* strains H37Rv and H37Ra in exponential phase at a 10 kb resolution. The color of the contact map, from white to red, indicates the log2 contact frequency. Axes indicate the genome position of each bin. (**B**) Exemplary snapshot of identified CIDs in *M. tuberculosis* strains H37Rv and H37Ra. CID separation scores and operon densities are plotted below the CID plot for both the strains.

The generated H37Rv and H37Ra interaction matrix exhibits two prominent diagonals as consistent with other bacteria (Fig. 1A). The two diagonals intersect each other at the center representing the terminus region (~2.2 Mb) and at corners representing the Ori region (0 and 4.4 Mb). This is consistent with the previous reports that the circular chromosome is organized in such a way that the origin and terminus occupy opposite poles of the cell and the chromosome is divided into left and right arms running along the axis (3, 18). The strong diagonal from the top left to the bottom right indicates that nearby loci were present on the same chromosomal arm and exhibit higher contact frequency. The less prominent diagonal from the bottom left to the top right represents lower frequency contacts, for example, those between opposite arms of the circular genome. Substantial distances at a physical level separate these genomic loci pairs, but the Hi-C data suggest that they were in close proximity, enabling their interactions in cellular context. We then compared the contact maps of H37Rv and H37Ra to examine the differences in their chromosome organization best studied at 10 kb resolution in this work (Fig. 1A). We observed a global similarity in the interaction map of H37Rv and H37Ra suggesting that the overall shape of chromosome remained conserved across these variants. Yet, critical differences were observed as discussed below.

### The virulent strain has a higher number of CIDs than the attenuated strain

In order to find the differences between the chromatin organization of CIDs in H37Rv and H37Ra, we employed domain caller program hicFindTAD (Materials and Methods) to detect the CIDs from corrected Hi-C interaction matrices generated at a 10 kb resolution. Despite a similarity between interaction maps of H37Rv and H37Ra at a global level, there was a considerable alteration in the number of CIDs as indicated by changes in their genomic positions (Fig. 1B). Our analyses revealed that the genome of H37Rv is partitioned into a total of 15 CIDs (total genomic occupancy of 85.90%) with size ranging from 40 kb to 720 kb (Table S2), whereas the H37Ra chromosome comprised of 9 CIDs (total genomic occupancy of 81.70%) with size ranging from 90 kb to 970 kb (Fig. 1B) (Table S2). Strikingly, we found that the median CID size was significantly higher in attenuated H37Ra (280 kb) as compared with virulent H37Rv (220 kb) (Fig. S2A). While some of the CIDs were conserved in both H37Rv and H37Ra, others were altered as indicated by changes in their genomic positions. Besides the few unique CIDs (CID 1 in H37Ra and CIDs 1 and 2 in H37Rv) and similar CIDs (CIDs 2, 4, 5, and 8 of H37Ra with CIDs 3, 7, 8, and 10 of H37Rv), we observed that few larger CIDs in H37Ra (CID 3 and CID 9) were partitioned into two or more smaller CIDs in H37Rv (CIDs 4, 5, and 6 and CIDs 11, 12, 13, 14, and 15). Conversely, two CIDs (CID 6 and CID 7) in H37Ra coalesce into a larger “merged” CID in H37Rv (CID 9). Collectively, these studies provide an indication of differential structural organization of CIDs in virulent and attenuated *M. tuberculosis* strains. To eliminate the possibility that the differential CID organization could be because of differences in the total number of valid reads in H37Rv and H37Ra, we also generated contact maps and CID plots for H37Ra by using a similar number of reads as H37Rv and confirmed the robustness of observed differences (Fig. S3).

We further calculated the CID separation score for each CID boundary and plotted their summary for both H37Rv and H37Ra. We found that despite differential CID organization in virulent H37Rv and attenuated H37Ra strains, there was no significant difference in the strength of CID boundaries (Fig. S2B) indicated by their CID separation scores. We observed that operon density is higher at CID boundaries compared with that within the CIDs (Fig. 1B). Also, CID separation scores and operon densities show a similar pattern in both H37Rv and H37Ra. However, newly created CID boundaries in region 3,240–3,960 kb in H37Rv exhibit higher operon densities as compared with those in H37Ra. The *nrdHIEF2* operon, which includes *nrdH* (Rv3053c), *nrdI* (Rv3052c), *nrdE* (Rv3051c), and *nrdF2* (Rv3048c), plays an important role in chromosome duplication and DNA repair (19), and the alteration in expression of *nrdHIEF2* operon might impact the growth and survival of *M. tuberculosis*. Interestingly, *nrdHIEF2* operon is placed near the boundary in H37Rv within CID 13 (3,350–3,550 kb) (59 kb) whereas in case of H37Ra, this operon is placed within CID 9 around 177 kb away from the boundary (Fig. S4). Similarly, the NADH dehydrogenase type I operon, encoded by nuoAN, is present at 22 kb from the CID boundary in H37Rv while in case of H37Ra, this operon is present within the CID 9,273 kb away from the boundary.

### Robustness against replicates

We have seen above that the Hi-C biological replicates were highly correlated with a Pearson correlation coefficient > 0.92 between both H37Rv and H37Ra (Fig. S1). To examine if the correlations and differences are random or conserved across the replicates, we plotted CIDs for two biological replicates independently for both H37Rv and H37Ra and observed that most of the CIDs are repeatable between the replicates but variable in CID boundaries (see Fig. S11). It has been shown previously that between replicates, TAD structures share only 60% of their boundaries, suggesting that chromosome structure is not a static feature but remains variable even in identical cell populations (20). In order to eliminate this variability, for the final outcomes discussed above, we merged biological replicates into a consensus contact matrix and then called interacting domains. To compare the overlaps across strains, CID boundaries from each replicate were aligned to the nearest bin with a boundary in H37Rv (Table S6). We observed that four CID boundaries in H37Ra and five in H37Ra had an exact overlap in all three replicates. In many more cases, two of the three replicates had boundaries in the same genome-wide bins. However, only three CID boundaries occurred within the same genomic bin across strains for any replicate from H37Ra and H37Rv each. This clearly shows a dominance of robust boundaries within replicates, which are not preserved across strains.

### Chromatin loop formation exhibits differential preferences in the right and left arms of the chromosome in H37Rv and H37Ra

**Fig 2:**
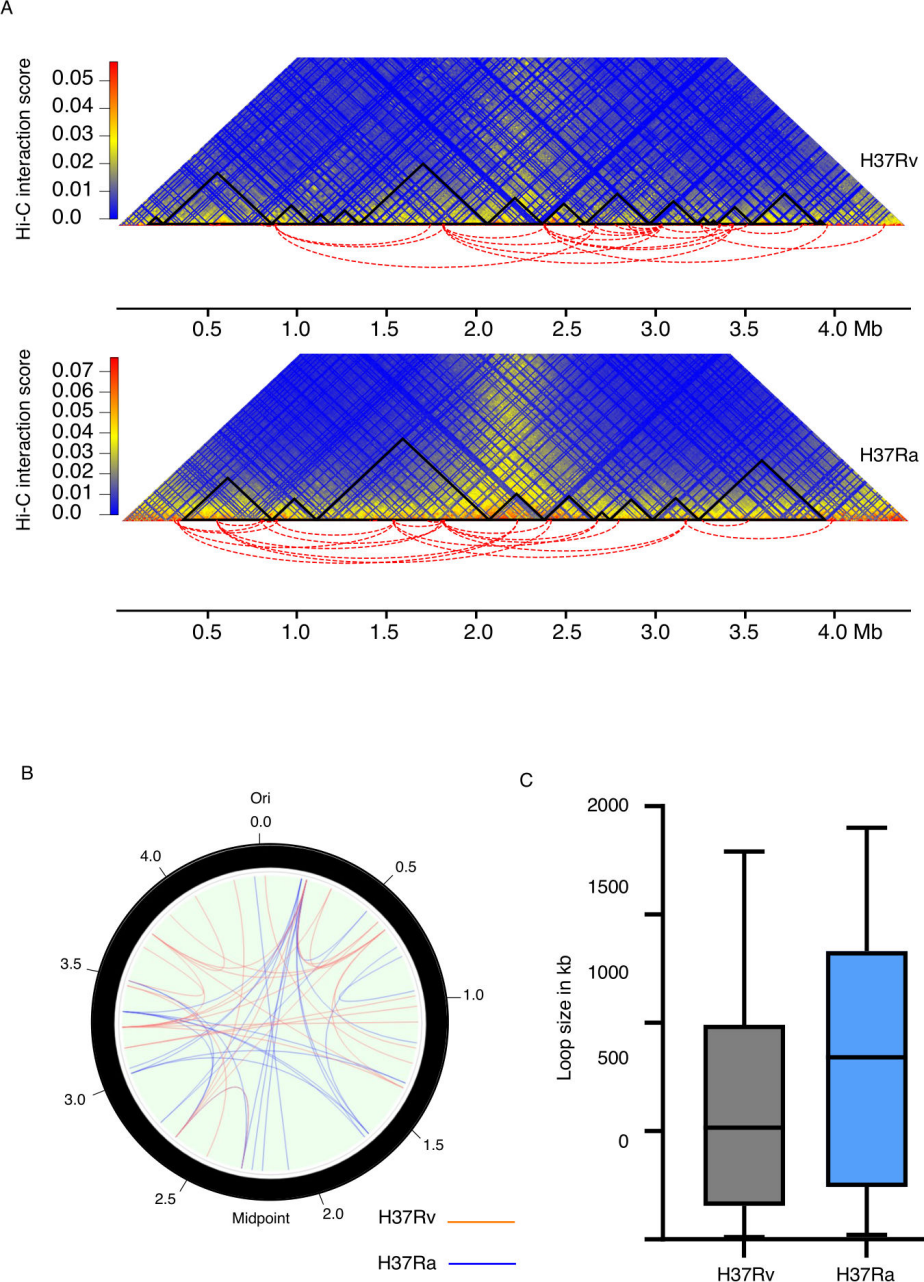
Chromatin loops identified in *M. tuberculosis* H37Rv and H37Ra genomes. (**A**) Long-range Hi-C loops identified using hicDetectLoops program of HiCExplorer in H37Rv and H37Ra. Arcs highlighted by red dotted lines represent the genomic positions of loops. (**B**) Circos plot of intra-chromosomal interactions in H37Rv and H37Ra (blue representing loops in H37Ra and orange in H37Rv). (**C**) Comparison of loop size in H37Rv and H37Ra (Wilcoxon signed-rank test, *P* > 0.05).

Chromatin loops bring distant regulatory segments into close proximity, thereby affecting their transcription (21, 22). DNA loop formation in bacteria is attributed to the NAPs such as H-NS, FIS, and bacterial SMC proteins (6, 23, 24). To detect long-range contacts in both H37Rv and H37Ra, we employed hicDetectLoops program of HiCExplorer. HicDetectLoops can detect enriched interaction regions based on a strict candidate selection, negative binomial distributions, and Wilcoxon rank-sum tests. The maximum genomic distance limits the candidate selection, which in this study is 2 Mb. We identified 26 loops in H37Rv and 24 loops in H37Ra. Although we did not observe much difference between the number of loop formations in H37Rv and H37Ra, yet most of the long-range loops formed in H37Rv were present on the left arm of the chromosome, that is, from region 2 Mb to 4 Mb whereas in case of H37Ra, most of the loops were formed in region 0.2 Mb to 2.5 Mb (Fig. 2A). We also observed that most of the loops were unique and very few loops were common in both the strains (Fig. 2B) (Table S3). The size of loops in H37Rv ranges from 10 kb to 1,790 kb whereas it ranges from 30 kb to 1,900 kb in the H37Ra strain. However, the median loop size (840 kb) was higher in case of H37Ra as compared with H37Rv (515 kb) (Fig. 2C).

### Creation of new CID boundaries in H37Rv in region 3,250–3,960 kb corresponds to highly expressed genes in H37Rv as compared with H37Ra

**Fig 3:**
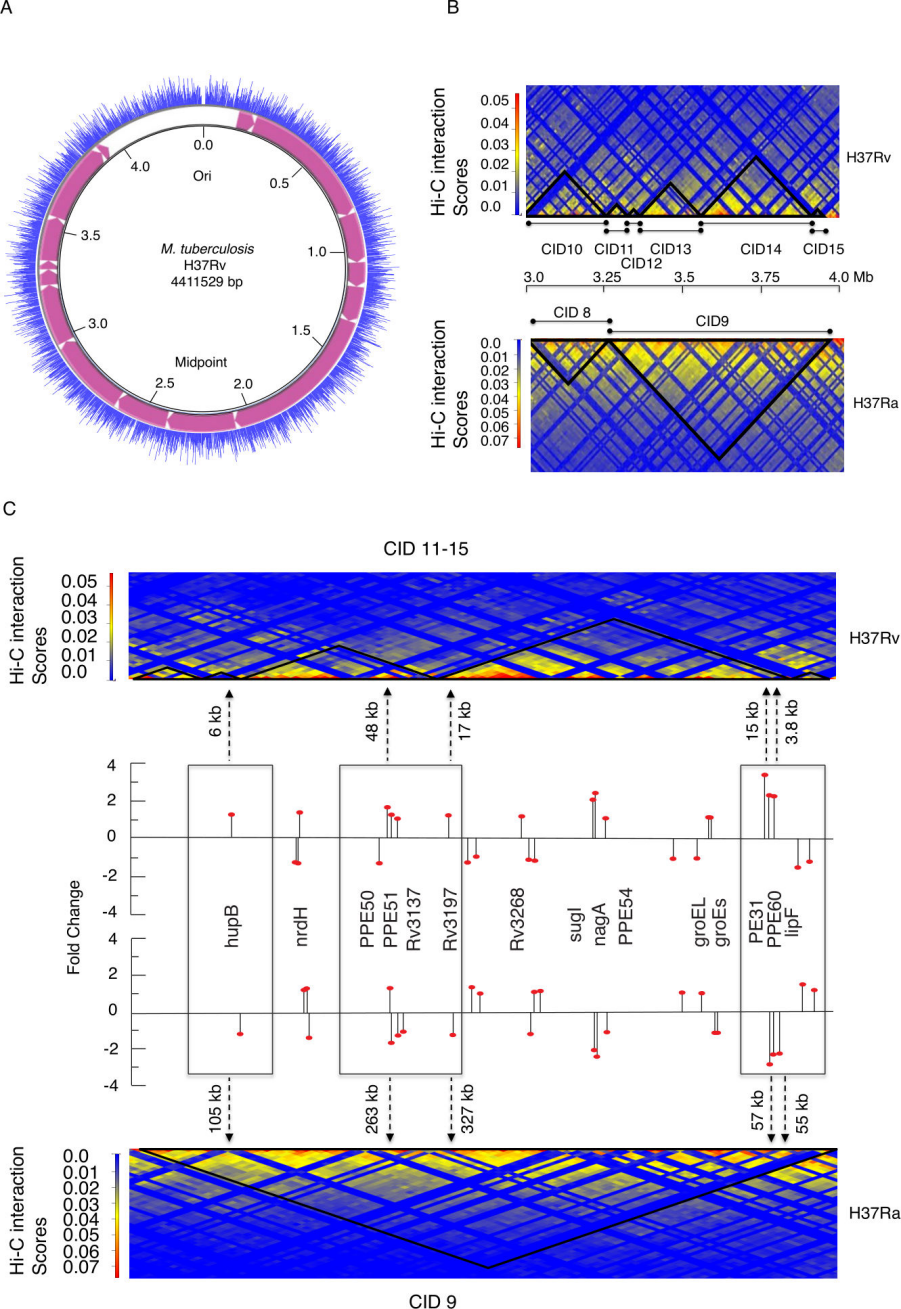
Highly expressed genes are present near the boundaries in H37Rv in a region corresponding to CID11–15. (**A**) Circular chromosomal map showing the gene expression (FPKM values) across the chromosome of *M. tuberculosis* strain H37Rv with the positions of CIDs indicated by pink color. (**B**) Comparison of presence of CIDs within the region 3,240,000–3,960,000 bp of *M. tuberculosis* chromosome. The upper and lower heatmaps represent H37Rv and H37Ra, respectively. (**C**) Plot showing genes with fold change expression (shown by black line) in H37Rv as compared with H37Ra on CID map corresponding to a region 3,240,000–3,960,000 bp for both the strains. The upper and lower heatmaps represent H37Rv and H37Ra, respectively. Black dotted line with arrow indicates the genomic distances of genes from the CID boundaries.

To find out the gene expression profiles along the CIDs, we created a circular chromosomal map of the *M. tuberculosis* H37Rv strain using the CGView tool and marked the position of CIDs within it. We plotted the average log FPKM score on the circular map of the H37Rv genome (Fig. 3A). We observed that most of the CID boundaries are enriched with highly expressed genes indicating a role of transcription in generating CID boundaries in *M. tuberculosis*. Similarly, we plotted average log FPKM on a circular map of the H37Ra genome and observed that most of the CID boundaries in H37Ra were also enriched with highly expressed genes (Fig. S5). This phenomenon seems to replicate highly expressed genes being located in nucleosome-free regions, widely observed in yeast and candida organisms (25). We further plotted the GC content over the CID maps of both these strains and observed that even though both H37Rv and H37Ra contain a GC-rich genome (65%), their few CID boundaries were marked with low GC content (Fig. S6). By comparative analysis of CID organization in H37Rv and H37Ra, we observed that CID 3 of H37Ra corresponds to CIDs 4, 5, and 6 of H37Rv. Similarly, CID 9 of H37Ra corresponds to CIDs 11, 12, 13, 14, and 15 of H37Rv (Fig. 3B) leading to a functional segregation for transcriptionally active zones. This differential organization in region 3,240–3,960 kb created new CID boundaries in H37Rv as compared with H37Ra. To understand whether the creation of new CID boundaries around the region 3,240–3,960 kb was consistent with the presence of highly expressed genes, we plotted genes with higher gene expression in H37Rv as compared with H37Ra on the CID map corresponding to region 3,240–3,960 kb for both the strains. (Fig. 3C). We used microarray data of differential gene expression of H37Rv and H37Ra available at GEO database (ID GSE7539) (26). The genes with significant differential expression in H37Rv and H37Ra are listed in a table with their genomic coordinates and CID locations (Table S4).

We found most of the genes like hupB, PPE50–PPE51, PE31, PPE60, and LipF having higher gene expression in H37Rv were present near the newly created boundaries corresponding to region 3,240–3,960 kb in H37Rv. The HupB gene is overexpressed in H37Rv (fold change 1.2) as compared with the H37Ra gene, and this gene is located 105 kb away from the boundary of CID 9 in H37Ra, but in case of H37Rv, creation of a new boundary at CID 11 (3,240–3,310 kb) placed this gene 6 kb away from the boundary. Similarly, PPE50–PPE51 genes with increased expression in H37Rv (fold change 1.68 and 1.28, respectively) were placed at 48 kb near the CID 13 boundary as compared with 263 kb away from the boundary in H37Ra. Similarly, PE31 and LipF genes that are overexpressed in H37Rv by fold change values of 3.3 and 2.5, respectively, are placed at 15 kb and 3.8 kb from the CID boundary in H37Rv. We also plotted genes with differential gene expression in H37Rv as compared with H37Ra on the CID map corresponding to region 1,070–2,070 kb for both the strains. It was observed that genes, which are overexpressed in H37Rv such as pks3, fadD21, PE13, and PPE18, were present near the newly created boundary between CID 5 and 6 in H37Rv (Fig. S7).

### CIDs corresponding to region 3,240–3,960 kb in H37Rv shows enrichment for different pathways suggesting functional segregation of domains

To understand whether this differential CID organization is playing any role in terms of pathway enrichment, we carried out pathway enrichment analysis of CID 9 of H37Ra and corresponding CIDs 11–15 of H37Rv using the ShinyGo tool (27). CID 9 of H37Ra did not show any enrichment; however, each of CIDs 11–15 of H37Rv showed enrichment for different biological pathways (Fig. S8). For example, CID 11 showed enrichment of pathways related to cell wall synthesis such as phthiocerol dimycocerosates (PDIMs), a group of complex lipids present in the *M. tuberculosis* cell envelope (28, 29). The PDIM cluster is organized in a separate CID, that is, CID 11 in H37Rv. Similarly, CID 12 showed enrichment of genes related to the carbohydrate metabolic process. Most of the pathways enriched in CID 13 are involved in oxidation-reduction processes including oxidative phosphorylation and ATP metabolic processes. CID 14 showed enrichment of genes related to the nucleoside metabolic process as well as carbohydrate metabolism whereas CID 15 showed enrichment of cholesterol catabolism-related genes. Collectively, it was observed that CIDs 11–15 of H37Rv, which correspond to CID 9 of H37Ra, showed enrichment of various different metabolic processes suggesting functional segregation of genes, that is, genes belonging to a particular metabolic process are clustered together.

### H37Rv CIDs in region 1,070–2,070 kb and 3,250–3,960 kb places differentially expressed PE/PPE genes near the boundaries

**Fig 4:**
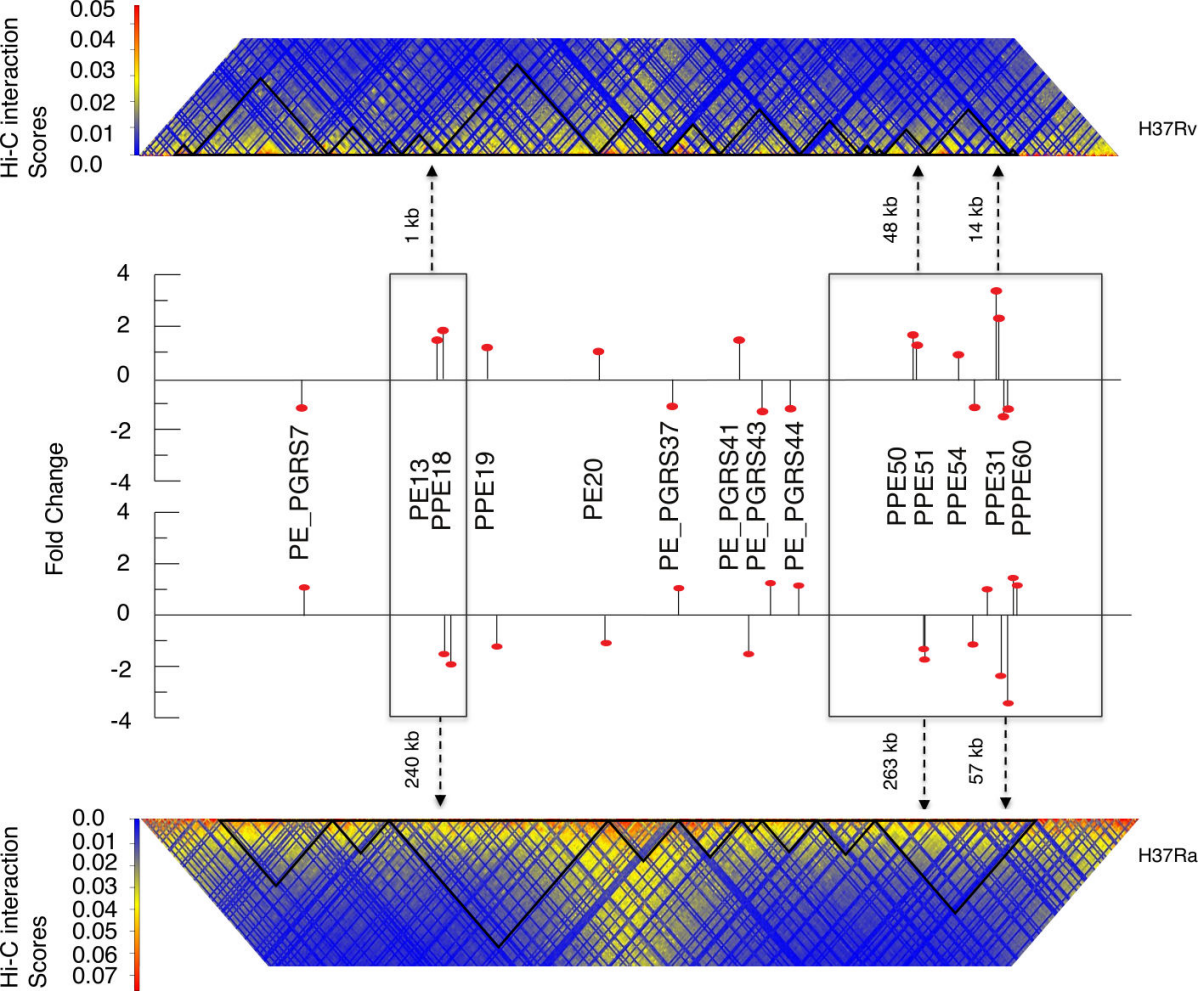
Differentially expressed PE and PPE genes are placed near CID boundaries in H37Rv as compared with H37Ra. Plot showing PE/PPE genes with fold change expression (shown by black line) in H37Rv as compared with H37Ra on CID map for both the strains. The upper and lower heatmaps represent H37Rv and H37Ra, respectively. Black dotted line with arrow indicates the genomic distances of genes from the CID boundaries.

The PE/PPE/PE-PGRS family of genes codes around 10% of the total genes of the mycobacterial genome (16, 30). The genes belonging to the PE/PPE family are distributed throughout the mycobacterium genome and are implicated in most diverse functions such as virulence, host cell binding, and the immune system evasion (31). To explore the possibility of variability of PE/PPE genes in H37Rv and H37Ra, we plotted PE/PPE genes with differential gene expression in H37Rv and H37Ra on the CID map of both strains (Fig. 4). We found that most genes belonging to the PE/PPE family of proteins were preferentially present near the CID boundaries in H37Rv but less prominently so in H37Ra (Table S5). Interestingly, most of the PE/PPE genes present near the CID boundaries have higher gene expression in H37Rv as compared with those in H37Ra. The PE13 and PPE18 gene pair is co-transcribed and is preceded by the ESX gene pair. It has been shown that reduced expression of the PE13 and PPE18 gene pair leads to attenuation of *M. tuberculosis* virulence (32). PE13–PPE18 genes were present within the interior region of CID 3 230 kb away from the boundary. But in H37Rv, this gene pair is moved 1 kb from the boundary region of CID 5. Similarly, PE31, a functionally important virulence gene, is overexpressed in H37Rv. This gene along with another PPE gene PPE60 is located 58 kb away from the boundary of CID 9 in H37Ra, but in H37Rv, this gene is placed 15 kb from the boundary of CID 14. Another gene belonging to this family PPE18 appears to be present in a cluster with ESAT-6-like proteins (33) and is one of the highly expressed genes in *M. tuberculosis*. Interestingly, PPE18 harbored one of those deletions in H37Ra, and its deletion contributes to virulence attenuation of *M. tuberculosis in vivo* (11). Interestingly, PPE18, which is located 240 kb away from the boundary of CID 3 in H37Ra, is moved near the boundary of CID 5 in H37Rv. So collectively, these findings suggest that the creation of new CID boundaries in H37Rv resulted in the positioning of these PE/PPE genes near the boundaries in H37Rv as compared with H37Ra.

### Physiological perturbation hypoxia causes CID condensation in virulent strain

**Fig 5:**
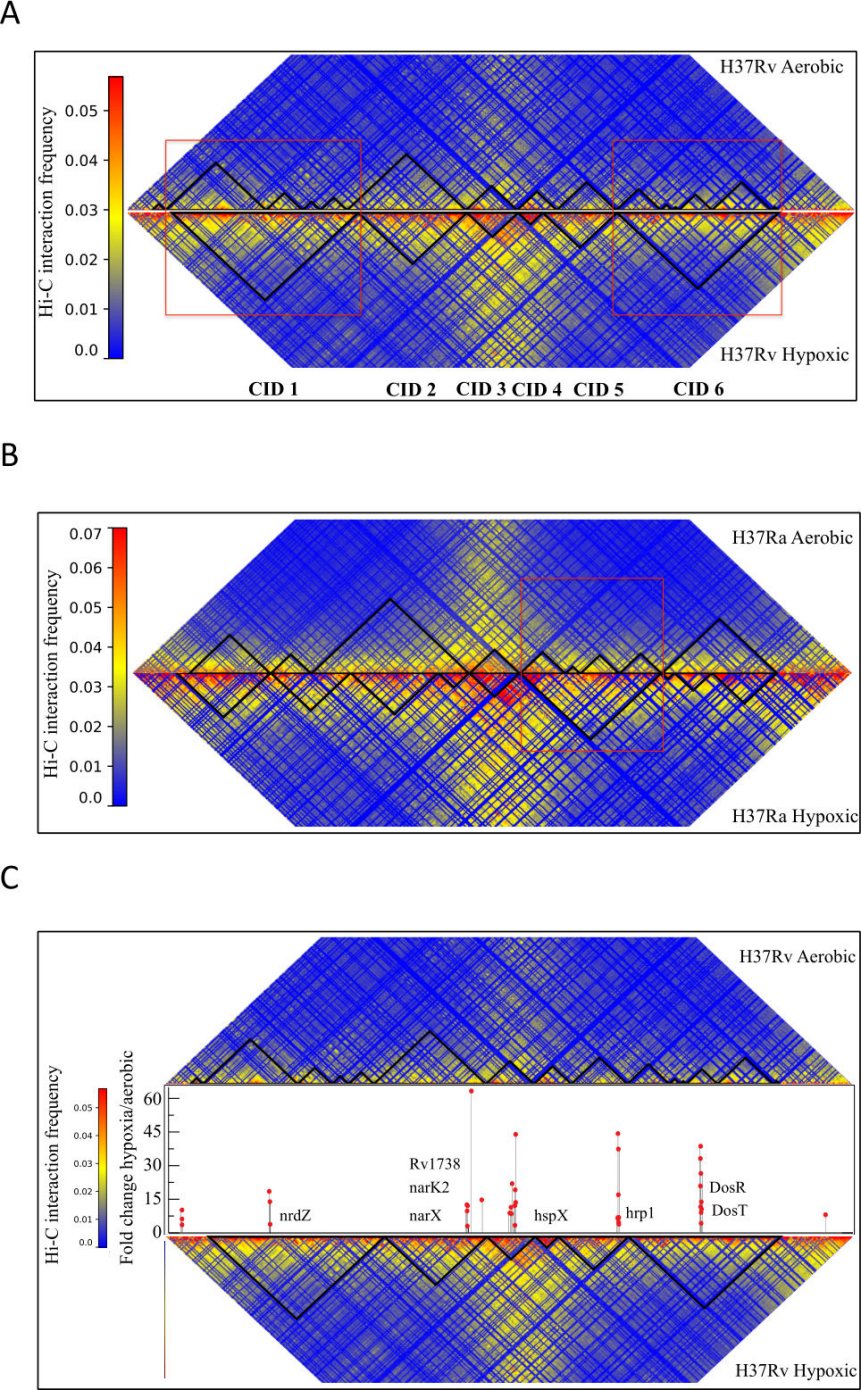
Comparative CID organization in *M. tuberculosis* H37Rv under aerobic and hypoxic conditions. (**A**) Comparative CID plots showing CIDs identified in *M. tuberculosis* H37Rv under aerobic (upper) and hypoxic (lower) conditions. Red boxes are highlighting the CIDs undergoing merging after hypoxia induction. (**B**) Comparative CID plots showing CIDs identified in *M. tuberculosis* H37Ra under aerobic (upper) and hypoxic (lower) conditions. Red box is highlighting the CIDs undergoing merging after hypoxia induction. (**C**) Comparative CID plots showing positions of DosR regulon genes along the CIDs identified in *M. tuberculosis* H37Rv under aerobic (upper) and hypoxic (lower) conditions with hypoxic/aerobic fold change expression (shown by black line with red dot).

The genome-wide interaction matrix generated for hypoxia-treated *M. tuberculosis* H37Rv cells was broadly similar to that of aerobic H37Rv cells, indicating that the hypoxia did not cause major changes in overall chromosome shape (Fig. S9A). We have observed similar results in the case of the H37Ra strain after hypoxia induction (Fig. S9B). However, the comparative analysis of H37Rv CID organization under aerobic and hypoxic conditions showed a remarkable reorganization of CIDs in the H37Rv chromosome after hypoxia induction (Fig. 5A). In particular, we found that the total number of CIDs was reduced to 6 in hypoxic-induced cells as compared with 15 CIDs observed in cells under aerobic conditions (Table S6). While some of the CIDs were conserved after hypoxia induction, most of the CIDs in hypoxic-treated cells were found to merge to form a larger CID. Further comparison of CIDs revealed that only a couple of the CIDs were conserved in H37Rv after its exposure to the hypoxic conditions and others were altered as indicated by changes in their genomic positions (Fig. 5A). CIDs 2, 3, 4, and 5 were observed to be merged into a larger CID within the region 240,000 bp to 1,400,000 bp after the hypoxia induction. Similarly, CIDs present in regions 2,970,000 bp to 3,950,000 bp (CIDs 10–15) were merged into a 980 kb large CID under hypoxic conditions. CIDs 8 and 9 were conserved with a single observation of rearrangement in the boundary region. These results indicate the existence of local chromatin reorganization in the *M. tuberculosis* H37Rv after hypoxia induction. Strikingly, we found that the median CID size was significantly higher in H37Rv (555 kb) under induced hypoxia as compared with H37Rv grown under aerobic conditions (220 kb).

A similar comparison of CIDs in H37Ra revealed that only one of the CIDs (CID 4) was conserved in H37Ra after its exposure to hypoxic conditions and others were altered as indicated by changes in their genomic positions (Table S7) (Fig. 5B). CIDs 2 and 3 were rearranged to form three CIDs within the region 850,000 bp to 2,070,000 bp after the hypoxia induction. Similarly, CIDs 5, 6, 7, and 8 were merged to form a larger CID within the region 2,380,000 bp to 3,250,000 bp under hypoxic conditions. CID 9 in H37Ra under aerobic conditions was divided into three smaller CIDs under hypoxia induction. These results indicate local chromatin reorganization in the *M. tuberculosis* H37Ra after hypoxia induction similar to that in H37Rv. We have also found a higher median CID size in H37Ra (410 kb) under induced hypoxia as compared with H37Ra grown under aerobic conditions (280 kb).

To investigate further molecular events in this regard, we decided to look at the DosR region of H37Rv. DosR regulon is a group of 48 co-regulated genes and has been shown to be induced under hypoxia in H37Rv in previous studies (34). To explore the possibility of the variability of DosR genes in H37Rv under aerobic and hypoxic conditions, we used microarray data of differential gene expression for H37Rv under hypoxic and aerobic conditions (34). We looked at the fold change in the expression of DosR regulon before and after hypoxia induction on the CID map of both strains (Fig. 5C). We found that apart from the gene cluster containing DosR and DosT genes, there were not many changes in the genomic positioning of other gene clusters belonging to DosR regulon with respect to CID boundaries.

**Fig 6:**
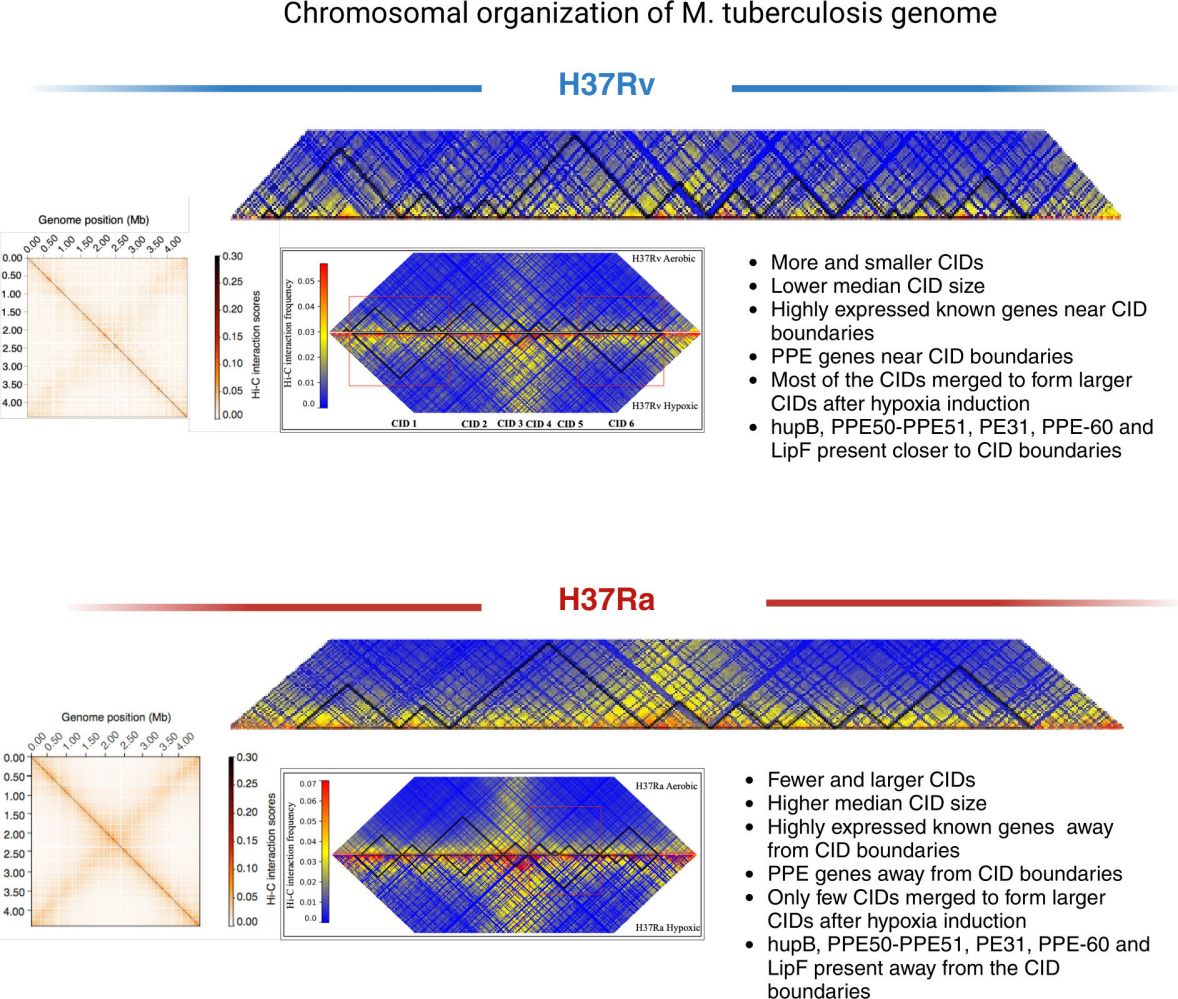
Graphical abstract. Schematic highlighting the distinctive features of chromosome organization in virulent H37Rv and avirulent H37Ra strains of *M. tuberculosis*.

Collectively, we have found that CIDs were organized differently in virulent H37Rv and avirulent H37Ra strains with the former exhibiting a lower median size. Also, most of the highly expressed genes and genes belonging to the PE/PPE family such as hupB, PPE50–PPE51, PE31, PPE60, and LipF were present closer to CID boundaries in virulent H37Rv as compared with the avirulent H37Ra strain. Furthermore, hypoxia induction caused a reorganization of CIDs in both H37Rv and H37Ra strains. The CID condensation upon hypoxia induction was found to be more profound in the virulent H37Rv strain leading to a larger media CID size as compared with that under normoxia condition (Fig. 6).

## DISCUSSION

Chromosome conformation capture-based methods such as Hi-C have been used in the past to study the bacterial genome organization and its role in transcriptional regulation. These studies in multiple bacteria such *E. coli*, *Mycoplasma pneumonia*, *C. crescentus*, and *B. subtilis* have indicated that their genomes were organized in 3D space as domains called CIDs similar to TADs in eukaryotic cells (1–5). However, the comprehensive understanding of how the multilayer organization of chromatin regulates strain-specific transcriptional activity remains unclear. Furthermore, the Hi-C data have been collected for a relatively small number of organisms and none for a variant of *M. tuberculosis*. Current work presented on the two canonical strains of *M. tuberculosis*, that is, H37Rv and H37Ra, analyzed individually and comparatively provided much needed insights into how the genomic packing in the two variants occurs, specifically and non-specifically.

A key finding presented above is that H37Rv and H37Ra strains of *M. tuberculosis*, which are very much similar in order of gene content, exhibit critically different 3D organization of their genome. Several studies have previously shown that eukaryotic genomes were partitioned into TADs of size in the range of 200 kb to 1 Mb (20, 21, 35–37). Our analysis allowed the detection of eukaryotic TAD-like domains (CIDs) for the first time in *M. tuberculosis*.

CIDs in both *M. tuberculosis* strains are larger than those previously reported in other bacteria such as *C. crescentus* and *B. subtilis* (3, 4). Our findings show that a substantial number of CIDs were conserved between H37Rv and H37Ra suggesting a robust and meaningful 3D topology in this family of bacteria across variants. Strikingly, however, we also found a number of CIDs that were appearing dynamic with structural alterations between H37Rv and H37Ra potentially providing for a previously unknown basis of their functional specificity. The major differences were observed in genomic regions corresponding to CID 3 (1,100–2,070 kb) and CID 9 (3,250–3,960 kb) of H37Ra. The corresponding genomic region in H37Rv showed the incidence of more numerous and smaller CIDs. The 710 kb region in H37Rv appeared partitioned into five CIDs (CIDs 11–15) compared with the corresponding region in H37Ra. A study performed by Boya et al*.* has earlier shown alteration in the structural organization of TADs during developmental transition from the pre-pro-B to the pro-B cell stage (38). They found that pro-B cells have a set of merged TADs generated due to coalesce of contiguous TADs present in pre-pro-B cells. However, the mechanism that regulates merging and splitting of CIDs remains to be understood. As both H37Rv and H37Ra have evolved from a same parental strain, this differential structural organization of CIDs during their evolution could be a possible factor for their differential pathogenesis. The presence of multiple insertions and deletion in H37Ra could be a possible driver of creation and abrogation of these CIDs during evolution.

Other than the numerosity of CIDs, the nature and frequency of chromatin loops are the other topological characteristics of genomic organization. In eukaryotic genomes, it has been shown that these loops bring distant regulatory segments into close proximity to regulate their transcription (21, 22). In eukaryotic cells, CTCF protein and the protein complex cohesin have been implicated in the formation and maintenance of these chromatin loops, establishing CTCF as the master weaver of eukaryotic genome (39, 40). Long-range chromatin loop formation is poorly studied in bacterial genomes. Even though proteins like CTCF and cohesin are missing from them, they do contain several NAPs such as H-NS, FIS, and bacterial SMC proteins that have been shown to participate in bacterial DNA looping (6, 23, 24). In our study, we did not observe significant differences between the number of long-range loops between H37Rv and H37Ra strains. However, interestingly, we did observe that the loops were present on different arms of chromosome in virulent and avirulent strains of *M. tuberculosis*.

Previously, CID boundary formation was correlated with the presence of highly expressed genes in *C. crescentus*. This way, the DNA is kept free of plectonemic loops during active transcription (3). Also, the formation of CIDs and TADs across organisms has so far revealed a number of differences between bacteria and mammalian chromatins (41). In some cases, CID/ TAD formation has been reported to be independent of transcriptional events or mediated by other factors such as histones or condensins. On the other hand, in bacterial genomes, active transcription has been reported to define CID formation. In such organisms, relocating active transcriptional cites has been shown to also relocate CID boundaries. The exact role of transcription in formation of CIDs and vice versa requires much more experimental work than envisaged in this current study, and hence, this question has not been addressed in the current work.

In this study, we have observed that CID boundaries in both H37Rv and H37Ra were marked by the presence of highly expressed genes. Apart from highly expressed genes, we also observed that low GC content levels characterize some of the CID boundaries. Similar reports in *E. coli* and *Salmonella typhimurium* and more recently in *B. subtilis* suggested that domain loop boundaries were localized in AT-rich regions (42, 43). Apart from prokaryotic organisms, it has been observed in the peanut genome too that TAD boundaries exhibit lower GC content as compared with the TAD interior region (44). Furthermore, the active chromatin compartment in the eukaryotic genome showed lower GC content and significantly higher transcription levels than the inactive compartment (44). Our results also revealed that most of the differentially expressed genes between virulent H37Rv and avirulent H37Ra were present close to the boundaries particularly in H37Rv and not in H37Ra. This observation is most pronounced in the regions corresponding to the 1,070–2,070 kb and 3,240–3,960 kb regions in both H37Rv and H37Ra. We found that these results may explain some of the poorly understood experimental observations in published literature. For example, HupB, an HU homolog, was recently identified in mycobacteria and it is one of the most abundant nucleoid-associated proteins. The HupB gene is differentially expressed in H37Rv and H37Ra and present near the CID boundary in H37Rv. It has been previously reported that deletion of the hu1 and hu2 genes in *C. crescentus*, which encode the HU1 and HU2 proteins, significantly decreased short-range interactions but did not affect global chromosome organization (11, 23). The role of HupB protein in domain organization in mycobacterium strains still needs to be elucidated, and the chromatin looping could be a potential reason for these observations.

Apart from placing highly expressed genes near the boundaries in H37Rv, the differences in CID organization within the region 3,240–3,960 kb between H37Rv and H37Ra also account for different enrichment of pathways in CIDs 11–15 in H37Rv. For instance, CID 11 in H37Rv showed enrichment of genes involved in cell wall synthesis (PDIM). Genes involved in PDIM biosynthesis are coded by a 70 kb gene cluster present within the region 3,240–3,310 kb, and interestingly, the size of CID 11 (3,240–3,310 kb) was also found to be 70 kb (16). This was remarkable in the fact that all genes involved in a similar pathway are clustered within a CID. Also within this cluster, fadD26, ppsA–E, drrA–C, and papA5 were also reported to form a single transcriptional unit (24). Similarly, CIDs 12, 13, 14, and 15 showed enrichments for different pathways, which suggest that the formation of these smaller CIDs in H37Rv might be playing a role in segregating genes involved in different pathways.

The PE/PPE/PE-PGRS family of genes contributes about one-tenth of the coding capacity of *M. tuberculosis*, and these genes are reported to be involved in various functions including virulence. A comparative genomic study between H37Rv and H37Ra discovered differences in genomic sequences of 35 PE/PPE/PE-PGRS genes. Furthermore, several PE/PPE/PE-PGRS genes found to be preferential “hot spots” for mutations (11). In our study, we found that most of the PE/PPE genes with higher gene expression in H37Rv were placed near the boundaries in H37Rv. Verma et al., 2017, observed that H37Rv and H37Ra are highly similar at the protein level. However, they observed marked differences in expression levels and phosphorylation patterns and could be responsible for the difference in their phenotypes. Significant differences in protein expression and phosphorylation in the PE/PPE/PE–PGRS gene family further support the genomic findings reported earlier (45).

We have also observed that physiological condition like hypoxia also induced notable changes in CID organization in the virulent strain, whereas in the avirulent strain, hypoxic condition did not induce significant CID reorganization. However, we did not observe significant changes in genomic locations of DosR regulon genes with respect to CID boundaries after hypoxia induction. The reorganization of CIDs in H37Rv after hypoxia induction may be providing one of the ways to regulate the expression of genes required for survival under hypoxic conditions.

Taking the above observations on CIDs and chromatin looping into account, we can conclude that even though H37Rv and H37Ra are very similar in DNA sequence, their 3D structure differs significantly. One would wonder how the sequence and structure of bacterial genomes come together to perform the biological function, including virulence. In particular, it would be interesting to know how small changes in sequences may lead to large-scale changes in CIDs and looping or local folding patterns of the DNA. This question becomes pertinent in view of topological changes between the two bacterial strains studied here, which appear to be driven by sequence-level alterations. How much sequence-level changes are sufficient to introduce topological changes remains a question to be explored. On the other hand, Hi-C provides a relatively lower resolution data and small and subtle changes in topologies that could occur at the local level in a sequence-dependent manner cannot be elucidated from these data. However, there is a body of evidence that shows that intrinsic sequence-dependent changes in a double-helical shape of the DNA can substantially impact transcriptional regulation, which can be explained only after translating the sequence signatures to their sequence-dependent DNA shapes (46). We have in the past shown that sequence-dependent conformational ensembles at the static and dynamic DNA shape levels can fill the gaps in the knowledge of target specificity of TFs (46). Due to the pioneering works from other research groups in this direction, it is now well known that shape signatures contained in genomic sequences are critical factors to consider for understanding their specificities. Thus, long-range DNA topological and local short-range DNA shape changes seem to emerge as the fundamental pillars of understanding functional differences between genomes beyond their sequences, and the current work focuses on the former.

Genome organization studies using Hi-C have proved to be useful in investigating the genome topology in multiple bacteria in the past. However, the role of genome organization in virulence has not been fully understood. This study demonstrated for the first time that virulent and attenuated *M. tuberculosis* strains exhibit distinct topological features that correlate with higher gene expression of virulence-associated genes in the virulent H37Rv strain. The distinctive features of chromosome organization in virulent H37Rv and avirulent H37Ra strains of *M. tuberculosis* were summarized in Fig. 6. Being the first attempt to solve the folding patterns of the *M. tuberculosis* genome, this study opens up new avenues for exploring strain-specific genomics of *M. tuberculosis* under physiological perturbation such as hypoxia. The Hi-C data sets presented here are expected to be useful in chromosome topology modeling and unraveling a new transcriptional regulatory network in *M. tuberculosis*.

## MATERIALS AND METHODS

### Growth conditions

*M. tuberculosis* strains were cultured in 7H9 medium supplemented with ADC enrichment (5% albumin, 2% dextrose, 0.003% catalase, and 0.85% sodium chloride) and 0.05% Tween 80. All the cultures were grown at 37°C with constant shaking at 200 rpm in a biosafety level 3 (BSL-3) facility. Optical density at 600 nm was measured to monitor growth.

### Hi-C library preparation

All experiments were performed in a BSL-3 facility. *H37Ra* and *H37Rv* chromatin was prepared as described previously with some modifications (16) (Fig. S10). Briefly, a total of 10^9^ cells were cross-linked with 1% formaldehyde and reaction was quenched with glycine. Cross-linked cells were lysed using lysozyme, and lysed cells were then digested using the BglII enzyme. After successful digestion, Biotin Fill-in was performed and cells were ligated under dilute conditions. DNA was isolated using the phenol chloroform method after proteinase K treatment. After removing biotin from unligated ends, Hi-C library was prepared using a NEBNext Ultra II DNA Library Prep Kit for Illumina and final DNA library was sequenced in the HiSeq Illumina platform. Sequencing reads were aligned, mapped, and filtered before generating a genome-wide contact matrix at 10 kb resolution using a HiCExplorer pipeline.

### Generation of the Hi-C contact map

HiCExplorer is a set of programs to process, normalize, analyze, and visualize Hi-C and cHi-C data, available on GitHub. Before using HiCExplorer to build a Hi-C contact matrix, paired-end reads were mapped, aligned, and filtered. Only valid Hi-C reads were used to generate contact maps at 10 kb resolution. Then, raw contact maps were normalized and corrected using the KR correction method, which balances a matrix using a fast-balancing algorithm introduced by Knight and Ruiz (2012) (47).

### Identification of CIDs

We used the hicFindTADs program of HiCExplorer, which uses a measure called CID separation score to identify the degree of separation between the left and right regions at each Hi-C matrix bin to detect the CIDs. We kept min depth at 30,000 bp and max depth at 60,000 bp with a step size equal to 10,000 bp.

### Loop detection

A program of HiCExplorer called hicDetectLoops was used for loop detection. hicDetectLoops detects enriched interaction regions (peaks/loops) based on a strict candidate selection, negative binomial distributions, and Wilcoxon rank-sum tests.

Experimental procedures including Hi-C library preparation, sequencing, and data analysis are described in details in Method S1.

## Data Availability

The Hi-C data have been deposited at the Gene Expression Omnibus (GEO) database (accession no. GSE218775).

## SUPPLEMENTAL MATERIAL

Supplemental figures msystems.00562-24-s0001.pdf Fig. S1-S13.

Legends msystems.00562-24-s0002.docx Supplemental figure legends.

Supplemental material msystems.00562-24-s0003.pdf Experimental details and supplemental tables.

Table S1 msystems.00562-24-s0004.xls Sequencing reads.

Table S4 msystems.00562-24-s0005.xlsx Genes with differential expression.

Table S5 msystems.00562-24-s0006.xlsx PE/PPE genes.

Table S6 msystems.00562-24-s0007.xlsx Robustness against replicates.

ASM does not own the copyrights to Supplemental Material that may be linked to, or accessed through, an article. The authors have granted ASM a non-exclusive, world-wide license to publish the Supplemental Material files. Please contact the corresponding author directly for reuse.
