# Supplementary material for: Differential genome organization revealed by comparative topological analysis of Mycobacterium tuberculosis strains H37Rv and H37Ra
Source: mSystems. 2025 Apr 7;10(5):e00562-24. doi: 10.1128/msystems.00562-24 (PMC12090813; doi:10.1128/msystems.00562-24)
Supplement: Legends — Supplemental figure legends. [file msystems.00562-24-s0002.docx]

**Supplemental legends:**

**Fig. S1. Pearson correlation between Hi-C replicates.** Pairwise scatter plots comparing interactions between three biological replicates in both H37Rv (Rv1, Rv2 and Rv3) and H37Ra (Ra1, Ra2, Ra3).

**Fig. S2. Comparison of CID size and CID separation scores in virulent and attenuated strains of *M. tuberculosis.* (A)** Box plot showing the median CID size (kb) in attenuated H37Ra and virulent H37Rv strains.
(B) Box plot showing the CID separation score in attenuated H37Ra and virulent H37Rv strains.

**Fig. S3. Contact map and CID map generated in H37Ra using similar number of reads as H37Rv.** Normalized BglII Hi-C contact map and snapshot of identified CIDs in *M. tuberculosis* strain H37Ra. The color of the contact map, from white to red, indicates the Log2 contact frequency. Axes indicate the genome position of each bin.

**Fig. S4**. **Comparison of Operon density between H37Rv and H37Ra.** CID plots showing Operon density distribution in H37Rv and H37Ra in a region from 3 to 4 Mb. Red box highlights the region showing region involving CID 12,13 and 14 of H37Rv.

**Fig. S5. Gene expression profile of H37Ra.** Plot of log FPKM scores (blue lines) on Circular chromosome map of H37Ra with marked CIDs (represented by pink arrows).

**Fig. S6. Plot of GC content on CID maps of H37Rv and H37Ra.** Circular maps of H37Ra and H37Rv with marked CIDs (represented by pink arrows). GC scores are marked on the CID on an outer ring represented by black lines.

**Fig. S7. Plot showing differentially expressed genes with fold change on CID maps of H37Rv and H37Ra**. Plot showing genes with fold change expression (shown by black line) in H37Rv as compared to H37Ra on CID map corresponding to a region from 1070000 to 2070000 bp for both the strains. The upper and lower heat maps represent H37Rv and H37Ra, respectively. Black dotted line with arrow indicates the genomic distances of genes from the CID boundaries.

**Fig. S8**. Go analysis of CIDs 11-15 in H37Rv.

**Fig. S9.** Normalized BglII Hi-C contact map of M. tuberculosis strain H37Rv (A) and H37Ra (B) under aerobic and hypoxic conditions at a 10kb resolution. The color of the contact map, from white to red, indicates the Log2 contact frequency. Axes indicate the genome position of each bin.

**Fig. S10. Quality control of Hi-C libraries**. (A) Integrity of Hi-C libraries was monitored by resolving Hi-C templates (100 ng) on a 0.8% agarose gel. All templates were visualized as a tight band of a size larger than 10 kb. (B) End fill-in efficiency of Hi-C libraries was monitored by amplifying a ligation junction formed by two BgIII restriction sites separated by a distance of 16 kb (amplicon 1) and 32 kb (amplicon 2). In Hi-C library successful fill-in and ligation of Bglll sites creates ClaI site, which is used to assess end fill-in efficiency of Hi-C libraries. (C) A schematic representation of designing of unidirectional primers for PCR digest assay.

**Fig. S11**. CID snapshots for two biological replicates independently for both H37Rv and H37Ra showing the most of the CIDs are repeatable between the replicates.

**Fig. S12**: Plot showing genomic insertion and deletions in H37Rv and H37Ra over their CID maps.

**Fig. S13**: Box plot showing comparison of operon density at CID boundary and non-CID boundary in both H37Rv and H37Ra.
