# Supplementary material for: Differential genome organization revealed by comparative topological analysis of Mycobacterium tuberculosis strains H37Rv and H37Ra
Source: mSystems. 2025 Apr 7;10(5):e00562-24. doi: 10.1128/msystems.00562-24 (PMC12090813; doi:10.1128/msystems.00562-24)
Supplement: Supplemental material — Experimental details and supplemental tables. [file msystems.00562-24-s0003.pdf]

**Supporting Information for**

# Differential Genome organization Revealed by Comparative Topological Analysis of *Mycobacterium tuberculosis* Strains H37Rv *and* H37Ra

Mohit Mishra, Ajay Arya, Md. Zubbair Malik, Akanksha Mishra, Rakesh Bhatnagar, Seyed E. Hasnain, Shandar Ahmad, Rupesh Chaturvedi

Rupesh Chaturvedi, Shandar Ahmad
Email: rupesh.chaturvedi.jnu@gmail.com, shandar@jnu.ac.in

**This PDF file includes:**

Supporting text
Figures S1 to S13
Tables S1 and S6
SI References

**Supporting Information Text**

## Hi-C experiments and data analysis

We performed Hi-C on exponentially growing cultures of Mycobacterium strains H37Rv and H37Ra after paired-end sequencing, the Hi-C reads are mapped, quality controlled and filtered using the Hi- C explorer (13).

**Methods**

## Chromosome conformation capture with next generation sequencing (Hi-C)

For each Hi-C experiment a total of $10^9$ cells were centrifuged at room temperature for 30 min at 5000 x g and re suspended in 20 ml Fresh 7H9 media. Cells were cross-linked with 1% formaldehyde by incubating at room temperature for 10 min. Crosslinking reaction was stopped by addition of 125 mM glycine followed by 10 min incubation on ice. Cross-linked cells were collected by centrifugation at 4°C for 20 min at 6000 x g and washed twice with 1X PBS. Cell pellets were resuspended in 1 ml of TE buffer (10 mM Tris pH 8.0, 1 mM EDTA) and cell suspension was transferred to a 2 ml eppendorf tube. 1/10 volumes of freshly prepared lysozyme (10 mg/ml) was added to each tube before incubating the tubes for 1 h at 37°C with constant shaking. 10% SDS was added to each tube at a final of concentration of 0.5% and tubes were incubated at room temperature for 15 min. Cells were centrifuged at room temperature for 20 min at 6000 x g. The supernatant was discarded and pellets were resuspended in 50 µl of TE buffer. Lysed cell suspension was transferred to 1.5 ml eppendorf tube and centrifuged at room temperature for 10 min at 6000 x g. Chromatin pellets were resuspended in 50 µl of TE buffer. Chromatin digestion buffer was prepared by adding 144 µl of nuclease free water, 25 µl 10% Triton X-100 and 25 µl of 10% NEB 3.1 buffer in a tube and 194 µl of digestion buffer was added to each tube. Tubes were incubated for at 37°C for 20 min with constant shaking. Next, 300 U of BglII (50 U/µl NEB) was added to each tube and tubes were incubated for 3 h at 37°C with agitation. To check digestion efficiency, 20 µl of sample was taken into a new tube before and after addition of enzyme and tubes were labeled as undigested and digested controls. 20 µl of TE buffer was added each time to make up the volume. 2 µl of proteinase k (20 mg/ml, Invitrogen) was added to each tube and tubes were incubated at 60°C for 20 min. DNA was extracted using phenol/chloroform/IAA (PCI) (sigma) and run on a 0.7% agarose gel. Another 300 U of BglII was added if digestion was not optimum and tubes were kept for another round of incubation. The next steps after successful digestion included labeling the DNA ends with biotin-dATP and performing blunt-end ligation of cross-linked fragments. This step allows ligation junctions to be selectively captured after the shearing. After successful digestion, tubes were briefly centrifuged and placed on ice. Biotin Fill-in master mix containing 10 mM dGTP, 10 mM dCTP, 10 mM TTP, 0.4 mM Biotin- dATP, 10X NEB buffer and 50 U of DNA Polymerase klenow fragment was added to each tube to fill in the overhangs generated after digestion. Tubes were mixed carefully and incubated for 45 min at 37°C. Tubes were placed back on ice and 10% SDS (0.5% final) was added to deactivate Klenow enzyme and tubes were incubated for 30 min at room temperature. Ligation mix containing 100 µl of 1x $T_4$ DNA ligase buffer (Invitrogen), 75 µl of 10% Triton X-100 (Sigma) and 5 µl of 20 mg/ml BSA (NEB) was prepared during the time of incubation and added to each tubes. Nuclease free water was added to each tube to make up final reaction volume to 1 ml. Ligation under dilute conditions was performed to promote intra-molecular ligation of cross-linked fragments. Tubes were mixed gently and incubated at 16°C for 20 min in a water bath. 10 µl of 5 U/µl T4 DNA ligase (thermo fisher) was added to each tube and tubes were then gently mixed by inverting several times before incubating at 16°C for 4 h with occasional mixing. 25 µl of 20 mg/ml Proteinase K was added to each tube and tubes were allowed to incubate at 60°C for 6 h. Tubes were allowed to cool at RT and divided into two 1.5 ml tubes. Equal volumes of phenol/chloroform/IAA (Sigma) were added to each tube. Tubes were vortexed for 1 min and then centrifuged for 10 min at RT. As much as possible aqueous phase containing DNA was transferred to a fresh tube and 1/10 volumes of 3 M sodium acetate buffer pH 5.2 (Sigma) were added to each tube. Content of the tubes was mixed by vortexing and 2 volumes of ice-cold 100% ethanol were added to precipitate DNA.

Tubes were mixed by inverting several times and then incubated at -80°C for at least 1h. Thereafter tubes were centrifuged at 15000 x g at 4°C for half an hour. DNA pellets were then washed twice with 70% ethanol and air-dried before re- suspending in 400 µl of TE buffer. Another round of purification was performed by doing one phenol/chloroform/IAA extraction and precipitating DNA by adding 1/10 volumes of 3 M sodium acetate, 2 volumes of 100% ethanol and incubating tubes at -80°C for 30 min. Thereafter tubes were centrifuged and DNA pellets were then washed twice with 70% ethanol and then air-dried before re- suspending in 50 µl of TE buffer. 1 µl of 1 mg/ml RNAse was added to each tube to degrade any RNA and tubes were incubated at 37°C for 30 min. 2.0 µL of the purified libraries were used to determine DNA concentration using the Qubit® dsDNA BR Assay Kit. Libraries were also visualized by running 100 and 500 ng of each sample on a 1% agarose gel. Presence of a relatively tight band with molecular weight >10 kb (for BglII Hi-C) indicated a good ligation (*SI Appendix*, Fig. S9).

**Biotin incorporation assay**

PCR was also performed to confirm amplification of a particular Hi-C junction. This amplified Hi-C junction is resistant to cutting by BglII but sensitive to digestion by ClaI (*SI Appendix*, Fig. S9). Sets of unidirectional primers (F and R) were used, which anneal to two neighboring BglII fragments (*SI Appendix*, Table S6). Primers are designed for two BglII fragments separated by a distance of 2 kb, 4 kb, 8 kb, 16 kb and 32 kb to measure the Biotin incorporation, PCR reactions were performed to amplify neighboring interactions in Hi-C and 3C samples as follows:

| Ingredient | 1 reaction |
|---|---|
| DNA | 100 ng |
| 10X PCR buffer | 5 µl |
| 10mM dNTP | 1 µl |
| Primer_1 (5µM) | 4 µl |
| Primer_2 (5µM) | 4 µl |
| *Taq* DNA polymerase | 0.66 µl |
| Water | To 50 µl |

**PCR conditions**

| Step | Temperature | Time |
|---|---|---|
| 1 | 95°C | 5 minutes |
| 2 | 95°C | 30 seconds |
| 3 | 58°C | 30 seconds |
| 4 | 72°C | 25 seconds |
| 5 | Go to 2 | 35 times |
| 6 | 95°C | 30 seconds |
| 7 | 58°C | 30 seconds |
| 8 | 72°C | 10 minutes |

## NGS library preparation

To remove biotin from unligated ends, 5 µg of Hi-C library was added to reaction mixture containing 1 µl of 10 mg/ml BSA, 10 µl of 10x NEBuffer 2, 1 µl of 10 mM dATP, 1 µl of 10 mM dGTP and 5 Units T4 DNA polymerase (NEB) in a total volume of 100 µl and incubated at 12°C for 2 h. 2 ml of 0.5 M EDTA pH 8.0 was added to stop the reaction. DNA was purified by phenol/chloroform/IAA and precipitated using ethanol. DNA pellets were then dissolved in 50 µl of TE buffer. 2000 nanograms of Biotin removed DNA from un-ligated ends was sheared on M220 Focused-ultrasonicator- Covaris by targeting 200-400 bp by following the conditions 20% Duty Factor, 200 cycles/burst, 50 W Peak Incident Power for 110 seconds at 4°C. Post to the fragmentation, size selection was performed using 0.7X to remove the fragments above ~400 bp and 1.2X to capture the fragments between 100-400 bp. An accurate fragmentation and size selection processes were confirmed by loading the size selected DNA into Tapestation D5000 screen tape. Further, the Biotinylated DNA fragments were pulled down using Streptavidin C1 beads and the sample was end repaired where in the buffer and the enzyme mix converts the overhangs resulting into blunt ends. The 3' to 5' exonuclease activity of end repair mix removes the 3' overhangs and polymerase activity fills in the 5'overhangs. To the blunt ended fragments adenylation was performed by adding single 'A' nucleotide to the 3' ends. To the adenylated fragments loop adapters were ligated and cleaved with uracil-specific excision reagent (USER) enzyme. The samples on the beads were further washed with buffer TLE and finally eluted in 20 µl of buffer TLE. To increase the library complexity, the above purified sample was proceeded for the amplification with 6 cycles in four reactions separately using NEBNext Ultra II Q5 master mix and NEBNext Multiplex Oligos for Illumina kit. The amplified products of all 4 samples were pooled and purified using 1.5X AMPure XP beads (Catalog: A63881, Beckman Coulter) to remove the fragments below 140 bp and the final DNA library was eluted in 20 µl of 0.1X TE buffer.

## Hi-C data analysis

After paired-end sequencing, the Hi-C reads are mapped, quality controlled and filtered using the Hi-C explorer (17).

## Reads Mapping

To construct the interaction maps of the *M. tuberculosis* H37Rv and H37Ra genome, read pairs were first uniquely mapped to the reference genomes of H37Rv (NC_000962.3) and H37Ra (NC_009525.1) covering 4411532 bp and 4419977 bp, respectively using bwa tool. With sequence match score is 1, penalty for mismatch is 4, gap extension penalty is 50; this is set very high to avoid gaps at restriction sites and produce better results as the sequences left and right of a restriction site are mapped independently. Penalty for 5' and 3'- end clipping is set to 0. Only read pairs for which both reads uniquely aligned to the genome were considered in subsequent steps. The H37Rv and H37Ra genomes were divided into restriction fragments (646 BglII fragments) and each read of a read pair was sorted into its corresponding restriction fragment. Read pairs were classified as valid Hi-C products, non-ligation products, or self-ligation products, and only the valid Hi-C products were subsequently considered below. After reads have been mapped the bam files are obtained for each individual mate reads using samtools.

## Creation of Hi-C Matrix

HiCExplorer is a set of programs to process, normalize, analyze and visualize Hi-C and cHi-C data, available on GitHub. We have used the HiCExplorer to build a Hi-C contact matrix and visualize it. The mapped reads have been used to build the Hi-C matrix. It is better to build the high-resolution matrix by keeping the low bin size (5Kb). Later we constructed the lower resolution matrix by merging higher resolution matrices into 10Kb bin size.

## Normalization of a Hi-C Matrix

In this normalization approach, matrix scores are scaled into the 0 to 1 range by selecting the "norm_range" normalization. However, it does not compute the contact probabilities. In this mode of normalization all read counts are set to the 0 to 1 range i.e. the maximum value of Hi-C matrix is 1 and minimum value of Hi-C matrix is 0.

## Correction of a Hi-C Matrix

The Hi-C matrix has to be corrected to remove GC content, open chromatin biases and most importantly to normalize the number of restriction sites per bin. Because a fraction of bins from repetitive regions contains few contacts it is necessary to filter those regions first. "KR" correction method is used to correct Hi-C matrices, it balances a matrix using a fast-balancing algorithm introduced by Knight and Ruiz (2012).

## Correlation analyses

Pearson correlation coefficients between Hi-C experiments were carried out as followed: two-dimensional matrices representing Hi-C contact maps were decomposed to one-dimensional vectors row-by-row. R was then used to compute the Pearson correlation coefficient between vectors.

## Identification of chromosomal interaction domains (CIDs)

We have used hicFindTADs program, which uses a measure called TAD-separation score to identify the degree of separation between the left and right regions at each Hi-C matrix bin. This is done for a running window of different sizes. Then, TADs are called as those positions having a local TAD-separation score minimum. The TAD-separation score is measured using the z-score of the Hi-C matrix and is defined as the mean zscore of all the matrix contacts between the left and right regions (diamond). To find the TADs, the program needs to compute first the TAD scores at different window sizes. Then, the results of that computation are used to call the TADs. We have used minDepth at 30000bp, and max depth at 60000bp with step size equal to 10000bp for computing CIDs in both H37Rv and H37Ra. Minimum boundary distance, which should be covered, is 30000, which is the flanking region of the bin size. correctForMultipleTesting, false discovery rate is selected for a multiple comparison with the threshold of 0.1.

## Loop detection

A program of Hi-C Explorer called hicDetectLoops was used for loop detection in both H37Rv and H37Ra. hicDetectLoops detect enriched interaction regions (peaks / loops) based on a strict candidate selection, negative binomial distributions and Wilcoxon rank-sum tests. The candidate selection is based on the restriction of the maximum genomic distance, here 2MB. For each genomic distance a continuous negative binomial distribution is computed and only interaction pairs with a threshold less than pValue preselection are accepted. In a second step, each candidate is considered compared to its neighborhood. This neighborhood is defined by the window size parameter in the x and y dimension. Per neighborhood only one candidate is considered, therefore only the candidate with the highest peak values is accepted. As a last step, the neighborhood is split into a peak and background region (parameter peak width). The peak Width can never be larger than the window size.

## Gene Ontology (GO) term association analysis on the genes present in CIDs in H37Rv and H37Ra

To determine whether the minor CIDs in H37Rv were enriched for particular GO terms ShinyGo tool was used (27). All the genes present in a particular CIDs were selected and all the genes present in whole genome were used as a background. Top 10 GO biological terms were plotted as a bar graph with the bar size representing the fold enrichment and color of the bar representing −log10 (p-value).

FDR is calculated based on nominal P-value from the hyper geometric test. Fold Enrichment is defined as the percentage of genes in your list belonging to a pathway, divided by the corresponding percentage in the background.

## Circular genome visualization with CIDs and GC content using CGView

The *Mycobaterium tuberculosis* H37Rv (NC_000962.3) and H37Ra (NC_009525.1) sequence files were downloaded from NCBI. CGView web server was used to create a circular genome map for both H37Rv and H37Ra (5). CIDs were marked on the circular lab by importing a text file containing genomic coordinates for each CIDs. The average GC content was plotted on the circular map using GC content plot tool.

## RNA-Seq data analysis

Raw RNA-sequence data of H37Rv (accession numbers SRX5057205 under BioProject PRJNA506544) and H37Ra (accession numbers SRX9339562 under BioProject PRJNA670515) wild-type strain was downloaded from the NCBI SRA database. All sequence data were assessed for quality control using FastQC. Resultant raw read data in the fastq format were initially processed with Trimmomatic [1], with low-quality reads being removed. The reference genomes used were Mycobacterium tuberculosis H37Rv (Accession no. NC_000962.3). Accurate alignment was executed using HISAT2 (version 2.1.0) [2]. Samtools (version 1.9) was used for the file format conversion required during the alignment and quantification steps. mRNA abundance was estimated by calculating FPKM values with cufflinks [3]. Differentially expressed genes (DEGs) were detected with a Cuffdiff (version 2.2.1) [3]. Cuffdiff was installed in Workstation Linux 8.0.7 environment. Circular genome mapping is constructed from ggplot2 in R software version 3.8.0. The graphs and figures were plotted using QtGrace [4].

## Plot of differentially expressed genes on CID map

We used microarray data of differential gene expression of H37Rv and H37Ra available at GEO database (ID GSE7539). We listed 110 genes, which showed significant differential gene expression between H37Rv and H37Ra. On the basis of their genomic coordinates, we categorized them into their respective CIDs. Then we plotted genes belonging to CID 9 of H37Ra and CID 11,12,13,14 and 15 with their fold change values using QtGrace.

**Table S2 List of chromosomal interaction domains (CIDs) in H37Rv**

| CID | Approximate Genome Position (kb) |
| --- | --- |
| 1 | 160-240 |
| 2 | 240-850 |
| 3 | 850-1070 |
| 4 | 1070-1180 |
| 5 | 1180-1340 |
| 6 | 1340-2060 |
| 7 | 2060-2370 |
| 8 | 2370-2610 |
| 9 | 2610-2970 |
| 10 | 2970-3240 |
| 11 | 3240-3310 |
| 12 | 3310-3350 |
| 13 | 3350-3550 |
| 14 | 3550-3910 |
| 15 | 3910-3950 |

**Table S3 List of chromosomal interaction domains (CIDs) in H37Ra**

| CID | Approximate Genome Position (kb) |
| --- | --- |
| 1 | 350-850 |
| 2 | 850-1100 |
| 3 | 1100-2070 |
| 4 | 2070-2380 |
| 5 | 2380-2660 |
| 6 | 2660-2750 |
| 7 | 2750-2990 |
| 8 | 2990-3250 |
| 9 | 3250-3960 |

**Table S6 List of chromosomal interaction domains (CIDs) in H37Rv (Hypoxia)**

| CID | Approximate Genome Position (kb) |
|---|---|
| 1 | 270-1400 |
| 2 | 1400-2060 |
| 3 | 2060-2370 |
| 4 | 2370-2520 |
| 5 | 2520-2970 |
| 6 | 2970-3950 |

**Table S7 List of chromosomal interaction domains (CIDs) in H37Ra (Hypoxia)**

| CID | Approximate Genome Position (kb) |
|---|---|
| 1 | 270-850 |
| 2 | 850-1340 |
| 3 | 1340-1880 |
| 4 | 1880-2070 |
| 5 | 2070-2380 |
| 6 | 2380-3250 |
| 7 | 3250-3330 |
| 8 | 3330-3560 |
| 9 | 3560-3960 |

**SI References**


1. Bolger, A. M., Lohse, M., Usadel, B. (2014). Trimmomatic: A Flexible Trimmer for Illumina Sequence Data. Bioinformatics 30 (15), 2114–2120.
2. Kim, D., Langmead, B., Salzberg, S. L. (2015). HISAT: A Fast Spliced Aligner With Low Memory Requirements. Nat. Methods 12 (4), 357–360.
3. Trapnell, C., Williams, B. A., Pertea, G., Mortazavi, A., Kwan, G., van Baren, M. J., et al. (2010). Transcript Assembly and Quantification by RNA-Seq Reveals Unannotated Transcripts and Isoform Switching During Cell Differentiation. Nat. Biotechnol. 28, 511–515.
4. Turner P (2005) XMGRACE, version 5.1. 19. Center for Coastal and Land-Margin Research, Oregon graduate Institute of Science and Technology, Beaverton, OR
5. Stothard P, Wishart DS (2005) Circular genome visualization and exploration using CGView. Bioinformatics 21:537-539
